# Exploring the Biological and Chemical Properties of Emerging 3D-Printed Dental Resin Composites Compared to Conventional Light-Cured Materials

**DOI:** 10.3390/ma18225170

**Published:** 2025-11-14

**Authors:** Nikola Živković, Stefan Vulović, Miloš Lazarević, Anja Baraba, Aleksandar Jakovljević, Mina Perić, Jelena Mitrić, Aleksandra Milić Lemić

**Affiliations:** 1 Department of Restorative Odontology and Endodontics, School of Dental Medicine, University of Belgrade, Rankeova 4, 11000 Belgrade, Serbia; nikola.zivkovic@stomf.bg.ac.rs; 2 Department of Prosthodontics, School of Dental Medicine, University of Belgrade, Rankeova 4, 11000 Belgrade, Serbia; aleksandra.milic@stomf.bg.ac.rs; 3 School of Dental Medicine, University of Belgrade, Dr Subotića Starijeg 8, 11000 Belgrade, Serbia; milos.lazarevic@stomf.bg.ac.rs; 4 University of Zagreb, School of Dental Medicine, Department of Endodontics and Restorative Dentistry, Gundulićeva 5, 10000 Zagreb, Croatia; baraba@sfzg.unizg.hr; 5 Department of Pathophysiology, School of Dental Medicine, University of Belgrade, Dr Subotića Starijeg 1, 11000 Belgrade, Serbia; a.jakovljevic@stomf.bg.ac.rs; 6 Research Laboratories, Implant-Research Center, School of Dental Medicine, University of Belgrade, Nebojšina 35, 11000 Belgrade, Serbia; 7 Institute of Molecular Genetics and Genetic Engineering, University of Belgrade, Vojvode Stepe 444a, 11000 Belgrade, Serbia; mina.peric@imgge.bg.ac.rs; 8 Institute of Physics, University of Belgrade, Pregrevica 118, 11000 Belgrade, Serbia; jmitric@ipb.ac.rs

**Keywords:** 3D-printed dental resin composites, biocompatibility, chemical stability, thermocycling

## Abstract

Advances in additive manufacturing have accelerated the development of 3D-printed dental resin composites. These materials contain a higher proportion of organic matrix and less filler than light-cured representatives, which may affect their behavior in the oral environment. This study aimed to evaluate the biological and chemical properties of 3D-printed dental resin composites before and after artificial aging, and to compare them with the light-cured representative. Specimens from a light-cured composite (Omnichroma—OMCR) and two 3D-printed composites (GT Temp PRINT—GTPR; SprintRay CROWN—SPRY) were subjected to aging treatments: unaged (T0) or thermocycled for 5000 (T1) and 10,000 cycles (T2). Biological evaluation was performed using MTT assay and Live/Dead cell fluorescence microscopy using human gingival fibroblasts, whereas Raman spectroscopy analysed materials’ structural changes. Materials exhibited good biocompatibility (>70% cell viability), with OMCR displaying greater variability. OMCR was more susceptible to chemical degradation under thermal stresses than both 3D-printed materials. Tested 3D-printed composites can provide comparable or even superior biological and chemical properties compared to light-cured representative, likely due to optimized resin formulations and post-curing protocols that improve polymer network organization and reduce residual monomer release. These findings support the potential of tested 3D-printed composites for manufacturing dental restorations.

## 1. Introduction

The emergence of three-dimensional (3D)-printed resins in restorative dentistry has significantly transformed clinical workflows, allowing for faster, more streamlined procedures and the fabrication of highly esthetic and functionally reliable indirect restorations. These materials, specifically developed for additive manufacturing, provide notable advantages in terms of customization, precision, and production efficiency. However, their unique composition, characterized by a higher proportion of resin matrix and lower filler content, raises certain concerns regarding biocompatibility, particularly related to the potential release of residual monomers. Maintaining the biocompatibility of dental materials is essential for preserving tissue integrity, supporting fibroblast activity, and ensuring patient safety [1]. Residual monomers released from composite restorations may diffuse through dentinal tubules toward the pulp or leach into the oral cavity [2]. When present in the oral environment, these compounds may enter systemic circulation through saliva or blood and exert systemic cytotoxicicity [3,4]. In addition, exposure to intraoral conditions, including fluctuations in pH, temperature, and moisture, induces physicochemical alterations in the resin, which may further affect its biological performance and compromise the long-term stability of restorations [5].

Although manufacturers rarely disclose detailed information about the resin matrix composition, filler morphology, or photoinitiator system, 3D-printed dental resin composites typically contain methacrylate-based monomers such as urethane dimethacrylate (UDMA), tetraethylene glycol dimethacrylate (TEGDMA), and 2-hydroxyethyl methacrylate (HEMA) [6]. These monomers have been shown to induce cytotoxic effects on human gingival cells [7,8], highlighting the importance of assessing the biological safety of these materials. The extent of monomer elution depends not only on the material’s chemical formulation but also on printing parameters, post-curing procedures, and the type of additive manufacturing technology employed [9]. Currently, stereolithography (SLA) and digital light processing (DLP) are the two dominant 3D printing techniques in dentistry [10]. SLA cures the resin point-by-point using a focused ultraviolet (UV) laser, whereas DLP polymerizes entire layers simultaneously using a digital projector [11] and has become the preferred 3D printing method for dental applications owing to its enhanced fabrication efficiency. Regardless of the printing method, achieving a high degree of polymerization is crucial to minimize the release of leachable components, including residual monomers and photoinitiators [12], which are primarily responsible for the cytotoxicity of resin materials. However, polymerization never reaches complete conversion, since some methacrylate double bonds (C=C) remain unreacted [13]. The final chemical integrity of polymerized resins is influenced by the filler-to-matrix ratio, resin formulation, and curing efficiency [5]. Studies have shown that nano-filled composites and materials with lower filler content exhibit altered polymerization kinetics and reduced structural stability [14]. This is particularly relevant for 3D-printed materials, which typically feature a lower filler load. Therefore, evaluating the chemical structure and molecular stability of such materials is fundamental for validating their clinical performance.

Although several in vitro studies have demonstrated acceptable mechanical performance of 3D-printed dental resin composites [15,16,17,18], data concerning their biocompatibility and chemical stability remain scarce, especially after exposure to simulated aging conditions. Therefore, the present study aimed to evaluate the biological and chemical properties of 3D-printed dental resin composites intended for temporary and permanent fixed dental prostheses (FDPs) before and after artificial aging, and to compare them with the light-cured representative. The null hypotheses were that: 1. There would be no significant differences in cell viability among tested materials before and after aging. 2. There would be no significant differences in chemical integrity among the tested materials before and after aging.

## 2. Materials and Methods

### 2.1. Specimen Preparation

**Figure 1:**
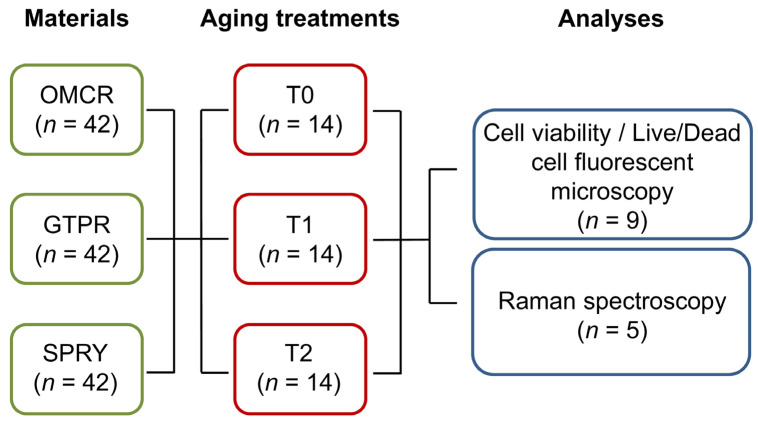
Number of specimens used in the study. OMCR—Omnichroma; GTPR—GC Temp PRINT; SPRY—SprintRay CROWN; T0—unaged; T1—aged for 5000 thermal cycles; T2—aged for 10,000 thermal cycles.

A total of 126 disc-shaped specimens (5 mm in diameter, 2 mm in thickness) were prepared from three dental resin composite materials (Table 1), with 42 per each material (*n* = 42) (Figure 1). The total specimen size was calculated using G*Power 3.1.9.7 software (Heinrich Heine University, Düsseldorf, Germany) according to the F test and “ANOVA: fixed effects, main effects, and interactions” considering large effect size 0.4, α 0.05, power 0.95, numerator df 4, and 9 groups (3 materials × 3 aging treatments).

Specimens of tested light-cured composite (Omnichroma, Tokuyama, Tokyo, Japan) (OMCR) were fabricated following a standardized conventional procedure. The material was inserted into a silicone mold (5 mm × 2 mm), covered with a Mylar strip (SS White, Philadelphia, PA, USA) and a microscope glass slide, then gently pressed to obtain a smooth surface and eliminate voids. Polymerization was carried out through the Mylar strip and glass for 20 s using a curing unit (Bluephase Style, Ivoclar Vivadent, Schaan, Liechtenstein) with an intensity of 1000 mW/cm^2^.

Specimens of 3D-printed composite for temporary (GC Temp PRINT, GC Europe, Leuven, Belgium) (GTPR) and permanent FDPs (SprintRay CROWN, SprintRay, Los Angeles, CA, USA) (SPRY) were designed to match required dimensions using software (Exocad 3.0, Darmstadt, Germany), exported as standard tessellation language (STL), and processed in another software (AccuWare V3.2.0.60, Shining 3D, Hangzhou, China). Printing was performed on a DLP printer (AccuFab-L4D, Shining 3D) under the following parameters: 90° build orientation, 50 μm layer thickness, and 405 nm wavelength. To prevent cross-contamination, a dedicated resin tray was used for each material. All 3D-printed specimens exhibited a solid internal structure that remained unchanged during the entire experimental procedure. After printing, the specimens were cleaned in 90% isopropyl alcohol using a cleaning system (FH-WA-01, Formlabs, Somerville, MA, USA) for 20 min, air-dried, and post-cured in a UV unit (FabCure 2, Shining 3D) for 30 min, following the manufacturers’ recommendations. Support structures were removed after curing.

All specimens were subsequently polished with silicon carbide abrasive papers (grits 600–1200, Buehler, Lake Bluff, IL, USA) under continuous water cooling for 20 s per side to ensure a uniform surface before further analyses. Final dimensions were confirmed using a digital caliper (Lukas Tools, Vogel, Kevelaer, Germany).

Before aging, all specimens were stored in distilled water at 37 °C for 24 h to stabilize the cross-linked polymer matrix and to remove residual unreacted monomers that could influence subsequent testing, in compliance with International Organization for Standardization (ISO) 7405:2025 [19] and previous research [20].

### 2.2. Aging Protocol

The specimens were randomly assigned to three independent aging treatments (*n* = 14) (Figure 1). In the first treatment, the specimens were unaged (T0). The specimens of the other two subgroups were subjected to thermocycling using a dedicated device (Thermo Cycler THE 1200, SD Mechatronik, Feldkirchen-Westerham, Germany). The procedure was adapted from the previously described protocol [21] and involved alternating immersion in cold (5 °C) and hot (55 °C) water baths, with a dwell time of 60 s in each bath and a transfer interval of 10 s between them, through 5000 (T1) and 10,000 cycles (T2) cycles. To minimize microbial growth, the specimens were rinsed with distilled water and placed in freshly prepared solutions every three days during the aging period.

### 2.3. Cell Viability

#### 2.3.1. Cell Culture

Evaluation of biological properties was carried out using primary human gingival fibroblast (HGF) cultures derived from three healthy donors (aged 18–22 years) who underwent surgical extraction of impacted mandibular third molars. The study was conducted in accordance with the Declaration of Helsinki, and approved by the Institutional Ethics Committee. Informed consent was obtained from all participants involved in the study.

HGFs were isolated using the explant technique [22]. Gingival tissue samples (approximately 3 mm^2^) obtained during surgery were immediately transferred to the cell culture laboratory in sterile containers. Samples were washed twice with phosphate-buffered saline (PBS) containing 1% antibiotic–antimycotic solution, finely sectioned with a sterile scalpel, and placed in T25 culture flasks. Cells were cultured in complete growth medium consisting of high-glucose Dulbecco’s Modified Eagle Medium (DMEM) (Gibco, Thermo Fisher Scientific, Waltham, MA, USA), supplemented with 10% heat-inactivated fetal bovine serum and 1% antibiotic–antimycotic solution. Cultures were incubated at 37 °C in a humidified atmosphere containing 5% CO_2_. The culture medium was renewed every 48–72 h, and cells were subcultured at approximately 80% confluence using 0.05% TrypLE (Gibco, Thermo Fisher Scientific) for 7 min at 37 °C. Cells from passages 3 and 4 were employed in all assays to ensure experimental reproducibility and minimize inter-donor variability. To confirm cell identity and purity, characterization of the isolated HGFs was performed using flow cytometry [23].

#### 2.3.2. Preparation of the Eluates

Within each group, nine specimens were designated for cell viability testing (*n* = 9) (Figure 1). Prior to testing, specimens were cleaned in an ultrasonic bath (US20, JSP, Chiyoda-ku, Tokyo, Japan) using 98% isopropanol for 2 min, followed by air drying under sterile conditions. Conditioned media were prepared in accordance with ISO 10993-12:2021 [24]. The surface area-to-volume ratio was standardized at 2.2 cm^2^ per 0.73 mL of complete growth medium. Specimens were incubated in complete growth medium at 37 °C in a humidified atmosphere containing 5% CO_2_ for 24 h to allow the leaching of potential cytotoxic components. The resulting eluates were collected and used for subsequent cell viability assays.

#### 2.3.3. MTT Assay

HGFs were seeded in 96-well plates at a density of 10,000 cells/well in 100 μL of complete growth medium and incubated for 24 h at 37 °C in a humidified atmosphere containing 5% CO_2_ to allow the formation of a semi-confluent monolayer. After reaching semi-confluency, the growth medium was replaced with the previously prepared composite eluates, and the cells were incubated for 24 h, 48 h, and 72 h, in accordance with ISO 10993-5:2021 [25] and previous research [26]. Before assessing cell viability, cell culture supernatants were collected at each time point and stored at −80 °C for subsequent analyses.

A 3-(4,5-dimethylthiazol-2-yl)-2,5-diphenyltetrazolium bromide (MTT) (Sigma-Aldrich, St. Louis, MO, USA) at a concentration of 5 mg/mL in DMEM (Gibco, Thermo Fisher Scientific) was used for determination of cell viability. MTT solution was added to each well and incubated for 4 h at 37 °C. The resulting formazan crystals were solubilized by adding 100 μL of dimethyl sulfoxide (Sigma-Aldrich), and absorbance was measured at 570 nm using a microplate reader (RT-2100C, Rayto, Guangming, Shenzhen, China). All experiments were performed in technical triplicate and independently repeated three times.

Cell viability (%) was calculated relative to the control (cells cultured in complete growth medium only, set as 100%) using the following formula:

$$Cell\;viability\;\left(\%\right)=\frac{\left(OD_{sample}-\;OD_{blank}\right)}{\left(OD_{PC}-\;OD_{blank}\right)}\times \;100$$

where OD represents the optical density, OD_sample_ corresponds to the absorbance of the test sample, OD_blank_ indicates the background absorbance, and OD_PC_ refers to the absorbance of the control.

The cytotoxic response to the tested resins was classified according to ISO 10993-12:2021 [24] as follows: non-cytotoxic (>90% survival), slightly cytotoxic (60–90% survival), moderately cytotoxic (30–60% survival), and severely cytotoxic (<30% survival).

### 2.4. Live/Dead Cell Fluorescent Microscopy

HGFs were seeded in 24-well plates at a density of 1 × 10^4^ cells/well and cultured in complete growth medium under standard conditions (37 °C, 5% CO_2_). After 24 h of initial incubation, the medium was replaced with the previously prepared materials specimens’ eluates, followed by additional 24 h incubation. Cell viability was evaluated using the Live/Dead cell imaging kit (488/570) (Cat. No. R37601, Invitrogen, Thermo Fisher Scientific), according to the manufacturer’s instructions. Briefly, the culture medium was removed, cells were rinsed with PBS, and then incubated in 2× staining solution for 15 min at room temperature. Fluorescence imaging was performed using a microscope (Axiovert 5, Zeiss, Oberkochen, Germany) equipped with an LD A-Plan 20×/0.35 objective lens and Fluorescein Isothiocyanate (FITC)/Tetramethylrhodamine Isothiocyanate (TRITC) filter sets to visualize live (green fluorescence) and dead (red fluorescence) cells. Cells cultivated in complete growth medium served as the positive control, whilst cells exposed to 96% ethanol for 5 min were designated as the negative control.

### 2.5. Raman Spectroscopy

To evaluate the initial chemical integrity of the tested materials and to monitor possible structural changes following artificial aging, Raman spectroscopy was performed on the surfaces of five specimens per each group (*n* = 5) (Figure 1). Measurements were carried out using a commercial NTegra Spectra system (NT-MDT, Moscow, Russia) equipped with a confocal optical microscope. A diode-pumped solid-state laser operating at 532 nm served as the excitation source. The laser power at the sample surface was set to 2 mW and focused within an area of approximately 0.5 × 0.5 μm^2^. Spectra were collected with an exposure time of 360 s per measurement, covering a spectral range of 400–1800 cm^−1^ with a spectral resolution of 2–4 cm^−1^. Prior to each measurement, the spectrometer was calibrated using the first-order silicon band at 520.7 cm^−1^. All spectra were recorded in an unpolarized configuration to minimize orientation-dependent spectral variations.

### 2.6. Statistical Analysis

Cell viability data were analysed using statistical software (SPSS 22.0, Chicago, IL, USA). The normality of data distribution was confirmed using the Kolmogorov-Smirnov test. Comparisons among materials within the same aging treatment, as well as comparisons among independent aging treatments within the same material, were performed using one-way analysis of variance (ANOVA), followed by Tukey’s post hoc test. Data were presented as mean ± standard deviation (SD). All *p* values of <0.05 were considered statistically significant.

## 3. Results

### 3.1. Cell Viability

**Figure 2:**
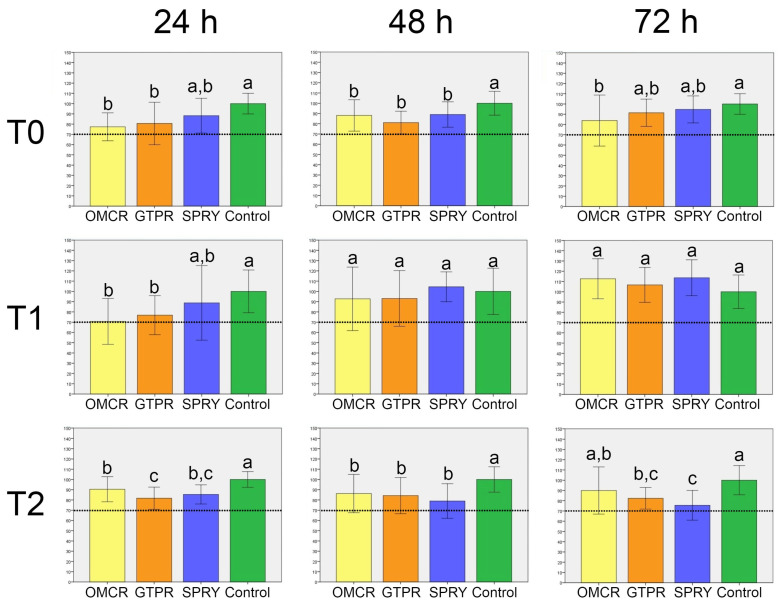
Cell viability (%) (mean ± standard deviation (SD)) of the tested materials (comparison among materials within the same aging treatment). OMCR—Omnichroma; GTPR—GC Temp PRINT; SPRY—SprintRay CROWN; T0—unaged; T1—aged for 5000 thermal cycles; T2—aged for 10,000 thermal cycles; Dotted lines—70% cell viability; Bars that do not share the same letter differ significantly from each other (*p* < 0.05; Tukey’s post hoc test).

**Figure 3:**
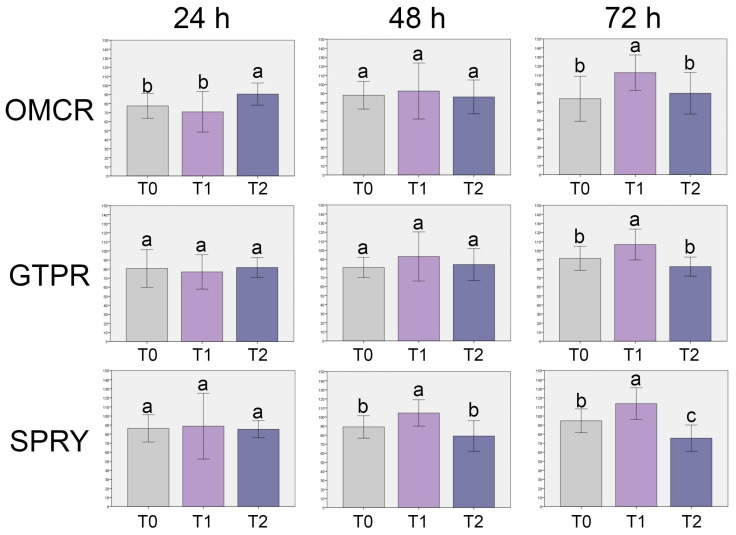
Cell viability (%) (mean ± standard deviation (SD)) of the tested materials (comparison among aging treatments within the same material). OMCR—Omnichroma; GTPR—GC Temp PRINT; SPRY—SprintRay CROWN; T0—unaged; T1—aged for 5000 thermal cycles; T2—aged for 10,000 thermal cycles; Dotted lines—70% cell viability; Bars that do not share the same letter differ significantly from each other (*p* < 0.05; Tukey’s post hoc test).

The results of the cell viability assay are presented in Figure 2 and Figure 3.

Regarding the comparison among materials within the same aging treatment (Figure 2), at T0, all tested materials exhibited cell viability above the biocompatibility threshold of 70%. OMCR showed the lowest viability among the tested materials, significantly lower than the control after 24 h (*p* < 0.05). GTPR and SPRY demonstrated slightly higher viability, but without statistically significant differences compared to OMCR (*p* > 0.05). Similar trends were observed after 48 h, with no significant differences among the materials (*p* > 0.05), although all values remained significantly lower than those of the control (*p* < 0.05). After 72 h, OMCR again showed the lowest viability and was the only material with significantly lower viability compared to the control (*p* < 0.05).

At T1, after 24 h, OMCR and GTPR exhibited significantly lower viability than the control (*p* < 0.05), while SPRY showed no significant difference (*p* > 0.05). After 48 h, viability increased in all materials, with no significant differences compared to the control (*p* > 0.05). This trend continued after 72 h, where all materials showed further increases in viability, exceeding control values, though differences remained non-significant (*p* > 0.05).

At T2, across all time points (24 h, 48 h, and 72 h), cell viability in all materials was significantly lower than in the control (*p* < 0.05), except between OMCR and control after 72 h (*p* > 0.05). Statistically significant differences were observed between OMCR and GTPR after 24 h, and between OMCR and SPRY after 72 h (*p* < 0.05).

Regarding the comparison among independent aging treatments within the same material (Figure 3), OMCR exhibited significantly lower cell viability at T0 and T1 compared to T2 after 24 h (*p* < 0.05), and at T0 and T2 compared to T1 after 72 h (*p* < 0.05). No other comparisons within OMCR showed statistically significant differences (*p* > 0.05).

For GTPR, significant differences were observed only after 72 h, where cell viability at T1 was significantly higher than T0 and T2 (*p* < 0.05). All other comparisons were not statistically significant (*p* > 0.05).

In the SPRY, cell viability was significantly higher at T1 compared to both T0 and T2 after 48 h and 72 h (*p* < 0.05).

### 3.2. Live/Dead Cell Fluorescent Microscopy

**Figure 4:**
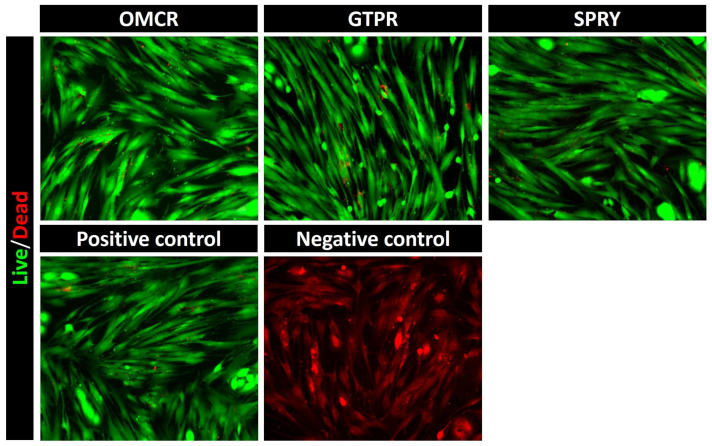
Live/Dead cell fluorescent microscopy analysis of human gingival fibroblasts (HGF) treated with tested materials’ specimen eluates. Representative micrographs were recorded at ×20 magnification. OMCR—Omnichroma; GTPR—GC Temp PRINT; SPRY—SprintRay CROWN.

Representative micrographs of fluorescent live/dead staining of HGFs incubated for 24 h with eluates from unaged tested materials’ specimens are presented in Figure 4. Micrographs of all tested materials showed extensive green fluorescence, indicating a high proportion of viable cells. The staining pattern closely resembled that of the positive control, which predominantly exhibited green-fluorescent viable cells, in contrast to the negative control micrograph showing uniform red fluorescence, characteristic of non-viable cell populations.

### 3.3. Raman Spectroscopy

**Figure 5:**
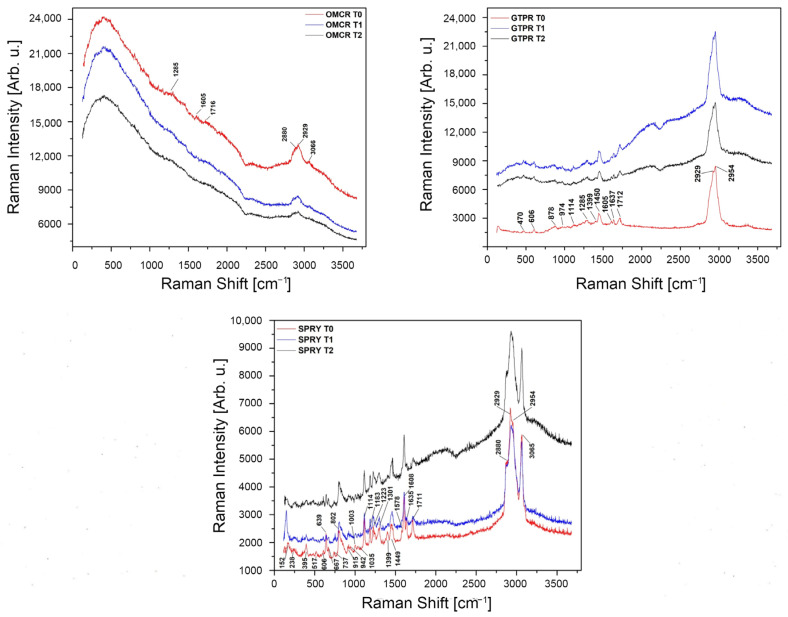
Raman spectra of the tested materials’ specimens. OMCR—Omnichroma; GTPR—GC Temp PRINT. SPRY—SprintRay CROWN. Each spectrum is consisting of three curves: red—T0 (unaged specimens), blue—T1 (specimens aged for 5000 thermal cycles), and black—T2 (specimens aged for 10,000 thermal cycles).

Raman spectra of the tested materials’ specimens are presented in Figure 5.

Raman spectra revealed distinct differences in the response of the investigated composites to thermal aging. Representative OMCR spectra demonstrated a progressive reduction in band intensity, particularly in the C–H stretching region (2880–3068 cm^−1^), accompanied by broadening of the carbonyl peak around ~1716 cm^−1^, suggesting high sensitivity to thermal aging. All major band assignments of OMCR samples are presented in Table 2.

Representative GTPR spectra initially exhibited increased intensity of characteristic bands after 5000 cycles, notably the C–H stretching doublet at 2929 and 2954 cm^−1^, as well as the aromatic and carbonyl peaks near ~1003 and ~1711 cm^−1^. Following 10,000 cycles, a partial reduction in intensity was observed in the carbonyl region, suggesting minor structural changes. All major band assignments of OMCR samples are presented in Table 3.

Figure 5 also depicts representative spectra of SPRY specimens before and after thermocycling, with key band assignments summarized in Table 4. The control spectrum displayed well-defined and intense bands across all major modes. Even after thermocycling, the C–H stretching and carbonyl bands remained sharp and prominent, with only minor intensity variations. Distinct aromatic (~1003 cm^−1^) and carbonyl (~1711 cm^−1^) peaks were consistently observed, indicating that SPRY maintained its chemical structure with negligible changes following aging.

## 4. Discussion

This study investigated the biological and chemical properties of light-cured and 3D-printed dental resin composites before and after artificial aging. Biological evaluation integrated cell viability of HGFs obtained through MTT assay and Live/Dead cell fluorescence microscopy to qualitatively validate the biocompatibility findings for eluates derived from unaged specimens. Chemical integrity, molecular and structural changes in the tested materials were analysed using Raman spectroscopy. This technique provided detailed insights into molecular vibrations, polymer chain organization, and bond stability within the resin matrix, making it a valuable tool for detecting structural rearrangements, degradation, and post-curing reactions [33]. The combination of biological and chemical assessment methods enabled a more comprehensive understanding of material performance after simulated aging. HGFs were selected as the cell model due to their high metabolic activity, anatomical proximity to restorative margins, prolonged exposure to dental materials, and key role in extracellular matrix remodeling and soft tissue repair. These features make HGFs among the first cells to interact with substances released from restorative materials, establishing them as a relevant and widely accepted in vitro model for assessing the biological response to dental resin composites [34]. In addition to HGFs, other cell types have been utilized in similar investigations, including human gingival keratinocytes [35], dental pulp stem cells [36], human leukocytes [37], as well as various fibroblast lineages [38,39]. The experimental timeline in this study was designed to assess the acute cellular response, with cell viability monitored daily for up to 72 h. Analyses were performed before (T0) and after thermocycling (T1 and T2). Thermocycling was employed as an in vitro aging procedure to simulate thermal fluctuations in the oral environment, ranging from 5 °C to 55 °C, thus reproducing clinical exposure to hot and cold stimuli [40]. The process was achieved through 5000 cycles to simulate six months (T1), and 10,000 cycles to simulate one year of clinical service (T2), according to previous studies [41,42]. Although thermocycling was conducted to more closely approximate intraoral conditions, some authors have argued that the applied temperature range may be excessively broad and not entirely representative of real clinical circumstances [21]. Conversely, accelerated aging protocols involving intensified environmental stressors are often used to estimate material longevity, as they provide insights into degradation kinetics under controlled conditions and allow extrapolation of the time required for the material to reach its failure point [40]. Additionally, thermally induced fatigue within polymer networks can cause several detrimental effects, including water sorption, polymer swelling, matrix breakdown, leaching of residual monomers, and molecular rearrangements. These phenomena may substantially impair biological behavior and chemical stability of the dental resin composites [43,44].

With regard to the cell viability, at T0, all tested materials demonstrated lower cell viability compared with the control, however, the values remained above the critical 70% threshold, indicating slightly cytotoxic behavior and, therefore, acceptable biocompatibility. Light-cured OMCR exhibited the lowest viability values at T0 after each 24 h, suggesting potential early-stage cytotoxic effects. Nevertheless, this was followed by recovery in later aging stages, particularly at T1, after 48 h and 72 h, reflecting the dynamic nature of material-cell interactions. These findings are consistent with previous research [45], where it was also observed an initial decline in cell viability for OMCR, followed by subsequent stabilization. This behavior may be associated with incomplete monomer-to-polymer conversion and ongoing in situ polymerization, resulting in a higher initial release of residual monomers. Among the 3D-printed composites, at T0, GTPR exhibited lower cell viability compared to SPRY, likely due to the synergistic effect of filler reinforcement and optimized post-curing parameters. However, both materials showed progressive improvement at T1, indicating increased biological stability. This pattern may be attributed to the early release of unreacted monomers, followed by continued post-curing polymerization and reduced leaching during storage, which collectively diminished cytotoxic effects. Similar results were reported in recent study [46], in which this phenomenon was linked to decreased monomer mobility within the polymer network over time, thereby limiting further release of residual compounds. At T1, the tested materials’ biological response stabilized, particularly after 48 h and 72 h. It seems that after 5000 cycles, the polymer matrix acted as a protective barrier, mitigating structural fatigue and minimizing the release of unreacted monomers. Interestingly, OMCR, that previously demonstrated significantly lower cell viability compared to control at T0 after each period, showed notable improvement following aging, even surpassing control value after 72 h. This observation is consistent with the findings of the previous research [44], which reported a progressive increase in the degree of conversion (DC) during the first 4000 thermal cycles of a commercial dental composite. The observed fluctuations in cell viability in OMCR, reflected by higher standard deviations at specific time points, may result from inherent biological variability or subtle differences in experimental conditions. Such variability highlights the necessity of incorporating multiple observation intervals and complementary analytical methods when evaluating the biological performance of dental materials. Following 10,000 thermal cycles (T2), all tested materials showed lower cell viability compared to control after each 24 h, 48 h, and 72 h periods; however, values remained above the established biocompatibility threshold. The reason for decreased cell viability at T2 might be the fact that repeated thermal expansion and contraction likely induced internal stresses and fatigue within the polymer matrix, leading to microstructural defects [47] that contributed to the delayed release of residual monomers from dental composites [48]. Among the 3D-printed composites, at T2, after 48 h and 72 h, GTPR exhibited slightly higher cell viability than SPRY for the first time, although the difference was not significant.

Live/Dead cell fluorescent micrographs confirmed good cell viability among all materials, with predominantly green-fluorescent viable cells observed in cultures exposed to eluates from all tested materials. The cell morphology and density were comparable to those of the positive control, indicating that the released substances did not induce cytotoxic effects or morphological alterations in HGFs. In contrast, the negative control displayed exclusively red-fluorescent, non-viable cells, thereby confirming the reliability and sensitivity of the assay. These results collectively demonstrate that tested materials exhibit favorable short-term biocompatibility profiles.

Considering all aforementioned, the first null hypothesis that no significant differences would be found in cell viability among tested materials before and after aging was rejected.

With regard to the evaluation of tested materials’ chemical integrity using Raman spectroscopy analysis, OMCR exhibited the greatest susceptibility to thermal stress, characterized by a gradual decrease in C–H stretching intensity and broadening of the carbonyl band that represent spectroscopic features commonly associated with polymer chain scission, oxidation, and weakened filler–matrix coupling [49,50,51]. These structural modifications indicate that OMCR may be more prone to degradation when subjected to clinically relevant temperature variations. In contrast, GTPR initially responded to 5000 thermocycles with an increase in C–H vibrational intensity, suggesting thermally activated secondary curing of residual double bonds. This phenomenon aligns with the transient improvement in cell viability observed at earlier intervals. The persistence of distinct C–H and C–O peaks indicates the formation of a denser and more cross-linked polymer network with restricted chain mobility [52]. However, prolonged exposure to thermal cycling (10,000 cycles) led to broadening of the carbonyl region and partial reduction of C=C bands, signifying the onset of hydrolytic degradation processes [53,54]. SPRY demonstrated higher structural stability, maintaining intense and sharp vibrational bands corresponding to aromatic and carbonyl groups, which remained largely unchanged after thermocycling. These results underscore the superior molecular integrity under simulated intraoral conditions of SPRY [55,56].

Considering all aforementioned, the second null hypothesis that no significant differences in chemical integrity among the tested materials before and after aging was also rejected.

The obtained findings suggest that properly processed 3D-printed composites can achieve comparable and even superior biological and chemical properties than conventional light-cured materials. Although the complete composition of the 3D-printed dental resin composites is mostly not disclosed, these materials usually contain common organic components such as UDMA and TEGDMA, whereas they differ mainly in filler type, content, and distribution. These variations, together with differences in polymerization mode, likely account for the distinct biological and chemical behavior of 3D-printed dental resin composites observed in the literature. In accordance with this statement, in the present study, despite the higher organic matrix fraction and lower filler content compared to light-cured representative, tested 3D-printed composites did not exhibit substantial degradation or monomer release. This stability may be attributed to a more homogeneous filler dispersion, which reduced the effective volume of the resin matrix and limited the proportion of unpolymerized monomers [57,58], as also seen in previous studies [59,60]. Furthermore, strict compliance with optimized post-curing protocols has been shown to markedly increase the degree of monomer conversion, enhance chemical stability, and minimize cytotoxic responses in gingival fibroblasts, ensuring cytotoxicity remains well below clinically relevant thresholds [46,61].

This study has several limitations that should be acknowledged. First, only one light-cured composite and two 3D-printed composites were evaluated. Therefore, the findings cannot be generalized to all restorative materials produced using these technologies but instead reflect the behavior of the selected products under the specific experimental conditions applied. Future investigations should include a wider spectrum of materials to better capture the variability associated with resin chemistry, filler composition, and manufacturing protocols. Likewise, the experiments were conducted under in vitro conditions using HGFs as a single-cell model. Although HGFs are relevant and widely accepted for assessing soft tissue responses, the absence of other biological factors, such as saliva, enzymes, oral microbiota, and immune components, limits direct extrapolation of the findings to the complex in vivo environment. Furthermore, the biological assessment was restricted to viability-based assays (MTT and Live/Dead staining), without inclusion of complementary endpoints such as oxidative stress generation, inflammatory cytokine release, or apoptotic signaling. Incorporating such analyses in future studies would provide a more comprehensive understanding of the cellular responses elicited by different materials. Moreover, the artificial aging simulation was limited to thermocycling at 5000 and 10,000 cycles, corresponding approximately to six months and one year of clinical function, respectively. While this approach is suitable for materials primarily designed for temporary restorations, extended aging protocols are required to evaluate the long-term durability of definitive restorative materials. Mechanical properties, including flexural strength, surface hardness, and wear resistance, were not examined, although these parameters are critical for predicting clinical performance under hydrothermal stress. Finally, chemical characterization relied exclusively on Raman spectroscopy. While Raman analysis provided valuable molecular-level insights into polymer network changes and degradation mechanisms, it cannot identify the degree of monomer conversion and leaching of unreacted components that may affect materials’ behavior. Therefore, additional analytical techniques, such as Fourier-transform infrared spectroscopy (FTIR), scanning electron microscopy with energy-dispersive X-ray spectroscopy (SEM/EDS), or X-ray photoelectron spectroscopy (XPS), would offer a more detailed characterization of surface morphology, elemental composition, and chemical bonding. Likewise, introducing the quantitative measurements of residual or released monomers in eluates may provide further insight into material stability and potential cytotoxic effects. Nevertheless, the findings of this study might be useful for clinical practice. The demonstrated favorable biocompatibility and chemical stability of 3D-printed dental resin composites indicate that these materials can be safely employed for provisional restorations and potentially for definitive restorations when processed under optimized post-curing protocols. Moreover, clinicians are advised to strictly adhere to manufacturer-recommended post-curing procedures to maximize polymer network conversion and minimize residual monomer release, thereby enhancing long-term performance and ensuring patient safety. However, future clinical studies are essential to confirm these findings.

## 5. Conclusions

Within the limitations of this study, the following conclusions can be drawn:

1. All tested materials demonstrated cell viability levels consistently above the established biocompatibility threshold. Tested light-cured composite showed the greatest variability in its biocompatibility profile, whereas the 3D printed composites exhibited a more stable biological response across all evaluation stages.
2. The light-cured composite displayed greater susceptibility to chemical degradation under thermal stress compared to both tested 3D-printed composites.
3. When properly processed, 3D-printed composites can offer comparable or even superior biocompatibility and chemical integrity compared to the light-cured composite. This is likely due to optimized resin formulations and post-curing protocols that promote improved polymer network organization and reduce residual monomer release, supporting their potential for clinical application in provisional and, possibly, definitive restorative procedures.

## Figures and Tables

**Table 1:** Materials used in the study.

Name	Fabrication	Code	Matrix	Filler	Filler Load	Manufacturer
Omnichroma	Light-cured dental resin composite	OMCR	UDMA, TEGDMA	Spherical SiO_2_-ZrO_2_ fillers	79 wt. %	Tokuyama, Tokyo, Japan
GC Temp PRINT	3D-printed dental resin composite for temporary FDPs	GTPR	UDMA (MMA free)	Silica nanoparticles	~20 wt. %	GC Europe, Leuven, Belgium
SprintRay CROWN	3D-printed dental resin composite for permanent FDPs	SPRY	UDMA, TEGDMA, other dimethacrylates	Ceramic nanoparticles	>50 wt. %	SprintRay, Los Angeles, CA, USA

3D—three-dimensional; UDMA—urethane dimethacrylate; TEGDMA—tetraethylene glycol dimethacrylate; MMA—methyl methacrylate; FDP—fixed dental prostheses.

**Table 2:** Raman bands assignations of Omnichroma (OMCR) specimens.

Raman Active Band [cm^−1^]	Assignment	Reference
1285	Asymmetric C–C–O stretching	[27]
1605	C=C stretching	[28]
1716	C=O stretching	[29]
2880	Symmetric CH_2_/CH_3_ stretching	[13,30]
2929	Asymmetric CH_2_/CH_3_ stretching	[28]
3066	=C–H stretching	[29]

**Table 3:** Raman bands assignations of GC Temp PRINT (GTPR) specimens.

Raman Active Band [cm^−1^]	Assignment	Reference
~470	Si–O–Si bending	[31]
606	Si–O/ring deformation	[13]
878	C–O–C stretching	[29]
974	C–O–C stretching	[29]
1114	C–O stretching of ester linkages	[28]
1285	Asymmetric C–C–O stretching	[32]
1399	CH_2_ scissoring/CH_3_ deformation	[13]
1450	CH_2_ scissoring/CH_3_ deformation	[13]
1605	C=C stretching	[28]
1637	C=C stretching	[28]
1712	C=O stretching	[30]
2929	CH_2_/CH_3_ asymmetric stretching	[30]
2954	CH_2_/CH_3_ asymmetric stretching	[30]

**Table 4:** Raman bands assignations of SprintRay CROWN (SPRY) specimens.

Raman Active Band [cm^−1^]	Assignment	Reference
238	Zr–O stretching	[31]
395	Mixed metal–oxygen	[31]
517	Si–O	[13]
606	Si–O	[13]
639	Si–O	[13]
667	Si–O	[13]
737	Si–O	[13]
802	C–O–C vibration	[29]
915	C–O–C vibration	[29]
942	C–O–C vibration	[29]
1003	Aromatic ring breathing	[30]
1035	C–O stretching	[28]
1114	C–O stretching	[28]
1183	Asymmetric C–C–O stretching	[32]
1223	Asymmetric C–C–O stretching	[32]
1301	Asymmetric C–C–O stretching	[32]
1399	CH_2_/CH_3_ deformation	[30]
1449	CH_2_/CH_3_ deformation	[30]
1578	C=C stretching	[28]
1608	C=C stretching	[28]
1635	C=C stretching	[28]
1711	C=O stretching	[13]
2880	CH_2_/CH_3_ asymmetric stretching	[30]
3065	=C–H stretching	[29]

## Data Availability

The original contributions presented in this study are included in the article. Further inquiries can be directed to the corresponding author.

## Abbreviations

The following abbreviations are used in this manuscript:

3D	Three-dimensional
DC	Degree of conversion
DLP	Digital light processing
DMEM	Dulbecco’s Modified Eagle Medium
FDP	Fixed dental prostheses
FITC	Fluorescein Isothiocyanate
FTIR	Fourier-transform infrared spectroscopy
GTPR	GC Temp PRINT
HEMA	2-hydroxyethyl methacrylate
HGF	Human gingival fibroblast
ISO	International Organization for Standardization
MMA	Methyl methacrylate
MTT	3-(4,5-dimethylthiazol-2-yl)-2,5-diphenyltetrazolium bromide
OD	Optical density
OMCR	Omnichroma
PBS	Phosphate-buffered saline
SEM/EDS	Scanning electron microscopy with energy-dispersive X-ray spectroscopy
SLA	Stereolithography
SPRY	SprintRay CROWN
TEGDMA	Tetraethylene glycol dimethacrylate
TRITC	Tetramethylrhodamine Isothiocyanate
UDMA	Urethane dimethacrylate
UV	Ultraviolet
XPS	X-ray photoelectron spectroscopy
